# Combining Health Information with Social Networking for LGBTQIA+ Older Persons

**DOI:** 10.1093/geroni/igaf122.2400

**Published:** 2025-12-31

**Authors:** Jennifer Smith, Sharon Bowland, Joseph Winberry, Namrata Mukherjee

**Affiliations:** University of Tennessee, Knoxville, Knoxville, Tennessee, United States; University of Tennessee, Knoxville, Tennessee, United States; The University of North Carolina at Chapel Hill, Chapel Hill, North Carolina, United States; University of Tennessee, Knoxville, Knoxville, Tennessee, United States

## Abstract

The aim of this research project was to address health-related concerns and missing information in the older LGBTQIA+ population in a community where historic discrimination and stigma made culturally responsive healthcare uncertain. We used surveys to collect data about healthcare needs and concerns during five social events held in a gay-friendly coffee shop over a six-month period. During the events, we invited providers to share essential health information on topics such as mental health, caregiving, and legal aid, to promote health literacy and to provide social connection. Fifty-three people over the age of 50 filled out the survey of likert scale and open-ended questions about their healthcare experiences. Forty-six percent of participants stated that they did not know where to find culturally supportive healthcare. One key theme was the difficulty participants identified in assessing whether providers were willing to provide services to LGBTIA+ people. On the other hand, participants identified a strong sense of connection with others in the LGBTIA+ community. Ninety percent said that they were linked with other LGBTIA+ people, and 100% indicated that they were actively involved in assisting others with their needs. Eighty-four percent said they also turned to their community for help. Participants seemed to value the opportunity to meet with others and the focus on their health and emotional well-being. Social groups that focus on health literacy and provide social outlets are using sustainable strategies for enhancing social connection and health information to bolster the LGBTQIA+ community and a sense of belonging.

